# β-Galactosidase-Catalyzed Transglycosylation of Tyrosol: Substrates and Deep Eutectic Solvents Affecting Activity and Stability

**DOI:** 10.3390/biom15060801

**Published:** 2025-05-31

**Authors:** Alžbeta Koššuthová, Monika Antošová, Vladena Bauerová-Hlinková, Jacob A. Bauer, Milan Polakovič

**Affiliations:** 1 Department of Chemical and Biochemical Engineering, Institute of Chemical and Environmental Engineering, Faculty of Chemical and Food Technology, Slovak University of Technology, Radlinského 9, 812 37 Bratislava, Slovakia; alzbeta.kossuthova@stuba.sk (A.K.); monika.antosova@stuba.sk (M.A.); 2 Department of Biochemistry and Structural Biology, Institute of Molecular Biology, Slovak Academy of Sciences, Dúbravská Cesta 21, 845 51 Bratislava, Slovakia; vladena.bauerova@savba.sk (V.B.-H.); jacob.bauer@savba.sk (J.A.B.)

**Keywords:** β-galactosidase, transglycosylation, phenylethanoid, deep eutectic solvents, thermal stability, enzyme activity

## Abstract

β-Galactosidase, a glycoside hydrolase enzyme, also possesses glycosyl transferase activity and can glycosylate various aglycones, including tyrosol, a phenylethanoid with antioxidant and health-promoting effects. This study examines the effect of lactose, tyrosol and deep eutectic solvents (DESs) as co-solvents on the stability and activity of *Aspergillus oryzae* β-galactosidase during the enzymatic synthesis of tyrosol β-d-galactoside (TG). The enzyme’s thermal stability was assessed using nanoDSF and circular dichroism spectroscopy, while the enzyme’s activity and specificity toward different glycosyl acceptors were investigated using the initial rate method. The effects of tyrosol and DESs on tyrosol galactoside synthesis over a 6 h period were also studied. Lactose and glycerol were found to stabilize the enzyme. Among the DESs tested, those containing betaine showed the highest stabilizing effect. The presence of DESs not only affected the overall enzyme activity but also changed the enzyme specificity, most frequently in favor of lactose hydrolysis. Components of DESs containing alcohol groups (polyols) also acted as transglycosylation acceptors. However, both glycerol and tyrosol were found to inhibit overall enzyme activity and TG synthesis. Overall, our findings provide new and valuable insights into the influence of reaction conditions on the stability and specificity of β-galactosidase.

## 1. Introduction

Tyrosol (2-(4-hydroxyphenyl)ethanol) is a biophenol occurring naturally in olive oil. Due to its antioxidant activity, it supports cellular antioxidant defenses [1] and exhibits neuroprotective effects [2]. Salidroside, the natural β-d-glucoside of tyrosol found in *Rhodiola* plants, is a promising compound with low toxicity and minimal side effects, exhibiting a broad spectrum of pharmacological properties [3]. Tyrosol β-d-galactoside (TG) demonstrates better neuroprotective effects than salidroside, likely due to differences in their glycosyl substituents [4]. Additionally, Kis et al. [5] reported that tyrosol β-d-fructofuranoside exhibits dose-dependent DNA-protective effects. Given that the pharmacological properties of tyrosol glycosides are influenced by their glycosyl moieties, further synthesis and investigation of biophenol glycosides are warranted.

The glycosidic bond in glycosides can be formed either chemically or enzymatically. While chemical synthesis is well developed, it is often labor-intensive and requires multiple protection and deprotection steps to prevent the formation of side products [6]. Additionally, chemical synthesis of glycosides can generate significant amounts of toxic waste. In contrast, enzymatic synthesis offers several advantages, including reduced environmental impact, lower energy consumption, and minimal waste generation, as well as high regioselectivity and stereoselectivity [7].

The synthesis of glycosidic bonds in nature is catalyzed by a group of enzymes known as glycosyltransferases (GTs). These enzymes mediate the biosynthesis of oligosaccharides and other glycoconjugates by transferring sugar moieties from donor molecules to suitable acceptors. GTs are broadly classified into two groups based on the type of glycosyl donor involved in the glycosylation reaction. The first group consists of Leloir pathway GTs, which utilize nucleotide sugars as donors and are capable of synthetizing polyphenol glycosides with high regioselectivity and yield [8,9,10]. While extensively studied as biocatalysts, their practical application is often limited by the low availability of GTs and high cost of both the enzymes and nucleotide sugars.

The second group comprises nonLeloir GTs, which use phosphorylated sugars or nonactivated di- or oligosaccharides as donors. For instance, the synthesis of the glucosides of hesperetin and epigallocatechin gallate was achieved using a commercial cyclodextrin glucanotransferase with starch as a donor [11,12]. This approach offers advantages such as low cost and readily available enzymes and carbohydrate sources, although it typically results in lower yields. An alternative to glycosyltransferases is glycoside hydrolases or glycosidases, which are a widespread group of enzymes present in metabolic pathways of all organisms [6,13]. Due to their broad availability and low cost, they are commonly used in various industrial applications.

Glycosidases hydrolyze the glycosidic bond between two or more carbohydrates or between a carbohydrate and a noncarbohydrate moiety. These enzymes can also catalyze the formation of glycosidic bonds via transglycosylation or reverse hydrolysis [14,15]. In reverse hydrolysis, only equilibrium concentrations of products can be achieved; therefore, transglycosylation yields depend strongly on kinetic factors. A key characteristic of glycosidases is their stereoselectivity toward the glycosidic bond being cleaved and their specificity for the remaining part of the sugar structure [15].

β-galactosidase (EC 3.2.1.23) is an enzyme exhibiting both hydrolytic and transglycosylation activities. While the former activity is widely exploited in the food industry to prepare low-lactose milk, the latter activity is utilized in the synthesis of galactooligosaccharides (GOS), which are important ingredients in prebiotic foods [16]. β-galactosidase from *Aspergillus oryzae* belongs to the GH35 family with the catalytic domain with conserved (α/β)_8_ TIM barrel fold [17]. This enzyme can galactosylate a wide variety of acceptors, including simple alcohols [18], salicin [19], polyols [20], and tyrosol [21,22].

Transglycosylation is a two-substrate reaction in which a disaccharide donor reacts with an enzyme to form an active enzyme–glycosyl complex. Various acceptors can bind to the glycosyl moiety, resulting in different products. If water acts as the acceptor, hydrolysis occurs, releasing a free monosaccharide. When the glycone donor serves as the acceptor, polymerization takes place, leading to the formation of oligosaccharides. If an aglycone is the acceptor, an aglycone glycoside is produced. These reactions occur simultaneously, and the maximal yield of each transglycosylation product depends on the relative rates of individual reactions. Since transglycosylation is typically a kinetically controlled process, the product concentration reaches a maximum at the point when the rate of hydrolysis begins to exceed the rate of formation [21].

Since hydrolysis is an undesirable side reaction in glycoside synthesis, the goal is to suppress it. This can be achieved either by using enzymes with high transferase activity and low hydrolase activity or by shifting enzyme specificity toward the desired products through a modification in reaction conditions. Key parameters include the concentration of the sugar donor and acceptor: higher saccharide concentrations reduce water activity, thereby lowering the rate of hydrolysis [23]. Additionally, the use of co-solvents can further decrease water activity and influence the enzyme’s specificity toward glycosyl acceptors. It is important to note that glycosidases have demonstrated activity in nonaqueous media; however, a minimal water content must be maintained, as water is essential for enzymatic activity [24]. Other advantages of using solvents in biocatalysis include increased solubility of hydrophobic substrates, suppression of water-dependent side reactions, and the ability to facilitate a broader range of reactions that are not feasible in aqueous environments [25].

Deep eutectic solvents (DESs) are a class of green solvents that were first described by Abbott et al. [26] in 2003. They offer an environmentally friendly alternative to conventional organic solvents. DESs consist of a hydrogen bond donor (HBD)—such as a quaternary ammonium salt—and a hydrogen bond acceptor (HBA)—such as sugars, polyols, or carboxylic acids— mixed at a eutectic molar ratio, at which the mixture becomes liquid at ambient temperature. Their low-cost precursors, simple preparation, non-volatility, non-flammability, and low toxicity make them an attractive option for various applications [27]. The use of DESs in biocatalysis has been shown to enhance enzyme performance.

For example, Xu et al. [28] found that β-glucosidase from *Aspergillus niger* exhibited a significant increase in activity in a DES solution. Additionally, the enzyme’s stability was higher in DESs than in buffer solution or methanol. The addition of a DES mixture composed of choline levulinate and ethylene glycol enhanced the lactose hydrolysis yield by β-galactosidase from *Kluyveromyces lactis* more than threefold [28]. However, the impact of DESs on enzyme activity is not easily predictable: while some DESs enhance enzymatic activity, others can completely inhibit it [29]. Furthermore, the positive or negative effects of a particular DES on the enzyme activity depend on the enzyme’s type and origin, as well as on the reaction conditions [28,30,31,32].

This study builds upon our previous investigations into the catalytic properties of β-galactosidase from *Aspergillus oryzae* [22,33]. It examines the effects of DESs and their individual components on the enzymatic activity and thermal stability of β-galactosidase during the transglycosylation of a therapeutically relevant biophenol. Thermal stability was assessed using nanoscale differential scanning fluorometry (nanoDSF) to evaluate the influence of lactose concentration, individual DES components, and complete DES mixtures. As a complementary technique, circular dichroism (CD) spectroscopy was employed to investigate the effects of lactose and tyrosol on β-galactosidase folding and stability. An initial set of reaction measurements was performed to assess the impact of DESs on both hydrolase and transferase activities of the enzyme during tyrosol transglycosylation. Additionally, the formation of tyrosol β-d-galactoside over an extended period was studied to evaluate the influence of DESs on product yield. The goal of this study was to advance the understanding of how substrates and DES co-solvents affect the stability and catalytic behavior of β-galactosidase, and further explore the applicability of DESs in transglycosylation reactions.

## 2. Materials and Methods

### 2.1. Materials

β-galactosidase from *A. oryzae*, Tegaferm LAC A100P, was purchased from Tegaferm Holding GmbH (Vienna, Austria). The activity of the enzyme determined according to the work of Adamíková et al. [33] with oNPG as a substrate was 174.0 ± 3.1 U/mg of enzyme powder and the protein content was 56% (*w*/*w*), determined by the Bradford method using bovine serum albumin as the protein standard. Tyrosol (98%) was purchased from Sigma-Aldrich Productions GmbH (Steinhem, Germany). Tyrosol β-d-galactoside (TG) standard for HPLC analysis was kindly gifted by Mgr. Elena Karnišová Potocká, PhD., from the Institute of Chemistry of the Slovak Academy of Sciences (Bratislava, Slovakia). Lactose, sodium carbonate, sodium acetate, acetic acid, glycerol, and ethylene glycol in purity pro analysis were purchased from Centralchem (Bratislava, Slovakia). Choline chloride (99%) and betaine (99%) were obtained from Carbosynth (Compton, UK). Choline acetate (98%) was purchased from Abcr (Karlsruhe, Germany). The Bio-La-Test Glu 500 enzymatic assay kit was obtained from Erba Lachema s.r.o. (Brno, Czech Republic). Acetonitrile and methanol, gradient-grade for HPLC analysis, and 1,4-butanediol were from Sigma-Aldrich Productions GmbH (Steinhem, Germany).

### 2.2. Preparation of Deep Eutectic Solvents

The DES components, hydrogen bond acceptors choline chloride (ChCl), choline acetate (ChAc), and betaine (Bet) were mixed with hydrogen bond donors’ urea (U), glycerol (Gly), ethylene glycol (EG), and butylene glycol (BG) in the molar ratios given in Table 1. The components were heated and continuously mixed at 80 °C in a water bath until a homogenous liquid was obtained. The DES–buffer solutions were prepared in acetate buffer with the desired DES concentration. Table 1 also shows the viscosities and densities of these DESs. The densities were measured at 25 °C on DMA 5000 Density Meter (Anton Paar, Graz, Austria). The viscosities were obtained from the literature.

### 2.3. Enzymatic Activity Assay

The overall, transglycosylation, and hydrolytic β-galactosidase activities were obtained using the initial rate of reaction method. The reactions were carried out in 4 mL of reaction media at 42 °C. The reaction mixture consisted of lactose and tyrosol dissolved in 0.1 M acetate buffer pH 5.5 with the addition of 10% (*v*/*v*) DES. The addition of some DESs altered the pH of the final reaction mixture; consequently, the pH of the acetate buffer was properly adjusted before DES addition to give a final reaction mixture pH of 5.5 ± 0.2. The concentration of lactose was 830 mM, and tyrosol concentrations were 36.2, 72.4, 144.8, and 289.5 mM. A 1 g/L β-galactosidase stock solution was prepared in 0.1 M acetate buffer and 10% (*v*/*v*) of DES and incubated overnight. 

To start the reaction, 50 μL of the enzyme solution was added to the reaction media. During the reaction, 50 μL samples were taken and immediately added to 150 μL of a 0.1 M Na_2_CO_3_ solution to inactivate the enzyme. The control sample (buffer-only) had the same substrate concentrations in 0.1 M acetate buffer, pH 5.5. The concentrations of substrates and products in the samples were determined using the analytical methods described in Section 2.7. The activity of the enzyme was defined as the amount of enzyme needed to convert 1 μmol of substrate (or produce 1 μmol of product) in 1 L of media per unit of time. The transglycosylation activities of the enzyme on tyrosol, lactose, and polyols were defined as the amount of TG, galactooligosaccharide trimer, and polyol galactoside (PGal) synthesized in 1 L of media in 1 s. The overall β-galactosidase activity was calculated from the initial rate of glucose release.

### 2.4. Nanoscale Differential Scanning Fluorometry (nanoDSF)

The thermal denaturation of β-galactosidase under various conditions (Table 2) was performed by nanoDSF using a Prometheus NT.48 (NanoTemper Technologies, Munich, Germany). The ratio of the intrinsic tryptophan (350 nm) and tyrosine (330 nm) fluorescence upon excitation with a 280 nm source was measured in real time. Measurements were performed over the range 20 to 95 °C in standard capillaries provided by the manufacturer (NanoTemper Technologies, Munich, Germany) using a thermal ramp of 1 °C per minute. The concentration of β-galactosidase in pH 5.5 0.1 M acetate buffer was 0.7 g/L; however, to attain good fluorescence in experiments involving tyrosol, the concentration was increased to 1.5 g/L. The enzyme stock solution was mixed with the test substance immediately before the experiment. When studying the DES components, the effect of the 24 h incubation period was examined and the thermal denaturation of the enzyme upon the addition of 10% (*v*/*v*) DES was measured. Melting points (T_m_) and onset temperatures (T_onset_) were calculated from the thermal curves using the stability analysis software PR.ThermControl v2.3.1 provided by the manufacturer.

### 2.5. Circular Dichroism (CD) Spectroscopy

The circular dichroism (CD) spectra of free β-galactosidase (0.02 g/L in 0.1 M acetate buffer, pH 5.5) were recorded on a Chirascan V100 spectrophotometer (Applied Photophysics, Leatherhead, UK) flushed with nitrogen at a flow rate of 5 L/min. The following parameters were used for recording far-UV spectra: spectral range of 280–190 nm, path length of 10 mm, spectral bandwidth of 3 nm, step size of 1 nm, and scan time of 1 s per point. Thermal unfolding was monitored at 42, 60 and 70 °C with the same spectral bandwidth and scan time. For each temperature, three repeats were measured and averaged. The recorded spectra were processed and averaged using Pro-Data Viewer from Applied Photophysics (Version 4.1.9) and the content of the secondary structure was determined by deconvolution with CONTINLL [38].

### 2.6. Enzymatic Synthesis of Tyrosol β-d-Galactoside

Enzymatic synthesis was carried out in the same way as the enzymatic activity assay but with a 5 g/L β-galactosidase stock solution. The reactions continued for six hours.

### 2.7. HPLC Analyses

The HPLC analyses were performed using an Agilent 1260 system using different columns for particular products, as described below. The HPLC system consisted of a vacuum degasser, a quaternary gradient pump, an autosampler with thermostated sample compartment, a column thermostat, a diode array, and refractometric detectors. The data were acquired and processed using Open Lab CDS ChemStation Edition C.01.10. The samples were suitably diluted in methanol or acetonitrile, depending on the column used, and filtered using 0.22 μm nylon syringe filters.

#### 2.7.1. Determination of Tyrosol and Tyrosol β-d-Galactoside Concentrations

The concentrations of tyrosol and TG were determined using a Zorbax Eclipse XDB C-18 column (150 × 4.6 mm, particle size 5 μm) with a guard column (12.5 × 4.6 mm) both from Agilent Technologies, Santa Clara, CA, USA. The mobile phase was an aqueous solution of 30% (*v*/*v*) methanol. The volumetric flow rate was set to 1 mL/min and the column temperature was 35 °C. The output was monitored using a diode array detector at a wavelength of 275 nm. Quantitative determination was carried out using the external standard method.

#### 2.7.2. Determination of Lactose, Galactooligosaccharide, Polyol and Polyol Galactosides’ Concentration

The concentration of lactose, GOS, and polyols was determined using two HILIC HPLC columns: Luna Omega Sugar column (150 × 4.6 mm, particle size 3 μm) from Phenomenex, Torrance, CA, USA, or Asahipak NH2P-50 4E (250 × 4.6 mm, particle size 5 μm) from Resonac, Tokyo, Japan. The mobile phase was 75% (*v*/*v*) acetonitrile in water with Luna Omega column or 68% (*v*/*v*) acetonitrile in water with Asahipak column. The mobile phase was 75% (*v*/*v*) acetonitrile in water. The volumetric flow rate was 1 mL/min and the column temperature was 35 °C in both cases. The output was monitored with a refractive index detector at 35 °C. Illustrative chromatograms are shown in Figure S1. Quantitative determination of lactose and polyols was carried out using the external standard method. Since a standard for GOS was not available, it was quantified using the parameters of the lactose calibration equation. The concentrations of polyol galactosides were calculated from the material balance of the corresponding polyol. Glucose and galactose were not separated using this column; therefore, glucose concentration was obtained using another analytical method and galactose concentrations were calculated from the material balance.

### 2.8. Determination of Glucose Concentration

The concentration of glucose was determined using the Bio-La-Test Glu 500 enzymatic assay kit. Thus, 10 μL was taken from the samples and mixed with 1 mL of the glucose test. These mixtures were then stored in a dark place for 30 min. Glucose concentration was quantified spectrophotometrically at a wavelength of 500 nm based on a calibration curve made from glucose standards.

### 2.9. Calculation of Material Balance and Activities

To properly calculate the initial rates of the ongoing reactions and the material balances, it was necessary to include reactions that can be independently evaluated:

(1) $${\mathrm{Lac}+H}_{2}O\overset{E}{\to }\mathrm{Gal}+\mathrm{Glu}$$

(2) $$\mathrm{Lac}+\mathrm{Tyr}\overset{E}{\to }\mathrm{TG}+\mathrm{Glu}$$

(3) $$\mathrm{Lac}+\mathrm{Lac}\overset{E}{\to }\mathrm{GOS}+\mathrm{Glu}$$

(4) $$\mathrm{Lac}+P\overset{E}{\to }\mathrm{PGal}+\mathrm{Glu}$$

Reaction (1) represents lactose hydrolysis, while Reactions (2) to (4) are transglycosylation reactions with different galactosyl acceptors. PGal is a polyol galactoside, which is the product of transglycosylation of a polyol P present in the DES. If there was no polyol or its transglycosylation was not observed, then Reaction (4) did not occur. The total concentration of released glucose $c_{Glu}$ was:

(5) $$c_{Glu_{1}}+c_{Glu_{2}}+c_{Glu_{3}}+c_{Glu_{4}}=c_{Glu}$$

where $c_{Glu_{1}}$, $c_{Glu_{2}}$, $c_{Glu_{3}}$, and $c_{Glu_{4}}$ are molar concentrations of glucose released from individual reactions (Equations (1)–(4)).

The concentration of galactose $c_{Gal}$ had to be calculated from the material balance. As follows from Equations (1) and (5),

(6) $$c_{Gal}=c_{Glu_{1}}=c_{Glu}-c_{Glu_{2}}+c_{Glu_{3}}+c_{Glu_{4}}$$

Since the concentrations of glucose released in Reactions (2) to (4) were equal to those of the other products, Equation (6) cand be rearranged as follows:

(7) $$c_{Gal}=c_{Glu}-c_{TG}-c_{GOS}-c_{PGal}$$

where $c_{TG}$, $c_{GOS}$ and $c_{PGal}$ are the molar concentrations of TG, GOS, and polyol galactoside.

The concentration of PGal was calculated as follows:

(8) $$c_{PGal}=c_{P,0}-c_{P}$$

where $c_{P,0}$ is the initial concentration of a polyol and $c_{P}$ is the concentration of a polyol at a given time. To check the consistency of the material balance, the molar concentrations of the glucosyl and galactosyl moieties were calculated:

(9) $$\sum c_{Glu}=c_{Lac}+c_{GOS}+c_{Glu}$$

(10) $$\sum c_{Gal}=c_{Lac}+2c_{GOS}+c_{TG}+c_{PGal}+c_{Gal}$$

The concentrations of both moieties were equal to the initial molar concentration of lactose:

(11) $$c_{Lac,0}=\sum c_{Glu}=\sum c_{Gal}$$

To calculate the enzyme activity in a particular reaction, the product concentration versus time was plotted, and the initial reaction rate, $r_{i}$ (in mM/s), was determined from the slope of the linear region. This procedure was applied to assess the rates of TG, GOS, and PGal formation. It could not be applied to determine the galactose formation rate. The relationship between the overall rates of glucose formation and lactose consumption and the rates of product formation is expressed by the following equations,

(12) $$r_{Glu}=r_{Gal}+r_{TG}+r_{GOS}+r_{PGal}$$

(13) $$r_{Lac}=r_{Gal}+r_{TG}+2r_{GOS}+r_{PGal}$$

Equations (12) and (13) were used to calculate the rate of galactose formation:

(14) $$r_{Gal}=r_{Glu}-r_{TG}-r_{GOS}-r_{PGal}$$

(15) $$r_{Gal}=r_{Lac}-r_{TG}-2r_{GOS}-r_{PGal}$$

The enzyme activities, $a_{i}$, were calculated according to the following equation:

(16) $$a_{i}=r_{i}\frac{V_{R}}{V_{E}}$$

where $V_{R}$ is the volume of reaction media and $V_{E}$ is the volume of the enzymatic solution added to start the reaction. To calculate the transglycosylation and hydrolytic activities of the enzyme, Equations (17) and (18) were used. Transglycosylation activity, $a_{trans}$, was calculated as the sum of the activities toward the formation of transglycosylation products TG, GOS, and PGal. The hydrolytic activity was calculated as the activity toward Gal formation. The total enzymatic activity is equal to the activity toward all ongoing reactions and can be calculated through the rate of glucose release.

(17) $$a_{trans}=a_{GOS}+a_{TG}+a_{PGal}$$

(18) $$a_{hydro}=a_{Gal}$$

(19) $$a_{total}=a_{Glu}$$

### 2.10. Statistical Analysis

Key measurements were repeated multiple times to ensure the experimental results were reliable and consistent. Time-course reactions and initial reaction rates were measured in duplicate, while stability measurements were conducted in triplicate for each sample to assess variability over time. Variability within and between samples was quantified using the STDEV.P function in Microsoft Excel for Microsoft 365 MSO (version 2504 Build 16.0.18730.20186). This function calculates the standard deviation based on the entire data population, providing a clear measure of dispersion for each set of repeated measurements. Differences between groups or experimental conditions were statistically evaluated using Student’s *t*-test, performed in Excel with the T.TEST function. The test compared the mean values between two groups to assess whether observed differences were statistically meaningful or occurred by random chance. All *t*-tests were conducted assuming a two-tailed distribution, with the choice of equal or unequal variances depending on the characteristics of the data. A *p*-value of less than 0.05 was considered statistically significant, helping to identify meaningful differences in parameters such as initial reaction rates or stability under different conditions.

## 3. Results and Discussion

### 3.1. Reaction Mechanism

**Figure 1:**
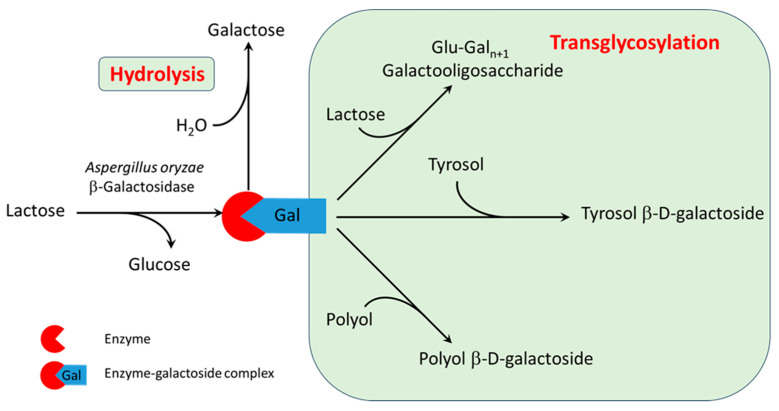
Reaction mechanism of transglycosylation of tyrosol and polyols catalyzed by β-galactosidase.

Based on the reaction products analyzed by HPLC, the mechanism of transgalactosylation catalyzed by β-galactosidase with different acceptors was identified, and it is illustrated in Figure 1. In the first step, lactose interacts with the enzyme to form an enzyme–galactoside active complex, releasing a free glucose molecule. In the second step, galactose is transferred from the active complex to the acceptor. When a water molecule acts as the acceptor, hydrolysis occurs, producing free galactose. Transglycosylation occurs when lactose or other aglycones present in the media (e.g., tyrosol or one of the polyols in some of the DES mixtures) serve as acceptors. This results in the formation of corresponding transglycosylation products, including galactooligosaccharides (GOS), tyrosol β-d-galactoside (TG) and β-d-galactosides of polyols. The stoichiometric equations of these reactions are provided in Section 2.8 (Equations (1)–(4)). The entire reaction system is kinetically controlled, as the rates of the individual reactions depend on the concentrations of the respective reactants.

### 3.2. Effect of Reaction Mixture Components on the Thermal Stability and Total Activity

The environment surrounding the enzyme affects its structure, stability, activity, and overall performance. In order to evaluate the effects of reaction mixture components on the enzyme, its thermal stability and activity under various conditions were measured. The thermal stability of the enzyme under various conditions was studied using nano differential scanning fluorometry (nanoDSF). This dye-free technique monitors changes in fluorescence as a function of temperature, which corresponds to the protein’s folding state [39]. As the temperature increases, the protein unfolds, and two important temperatures are determined: the melting temperature, T_m_, and the onset temperature, T_onset_.

T_onset_ is the temperature at which the first detectable changes in the protein’s fluorescence signal occur, indicating the beginning of the unfolding process. T_m_ is the temperature at which 50% of the protein is in the unfolded state. A higher T_m_ indicates greater protein stability under the given conditions. Environmental factors, such as pH, ionic strength, and the presence of specific anions or cations, can stabilize the protein through molecular interactions (e.g., hydrogen bonds, van der Waals forces) or by inducing conformational changes in the protein structure [40].

**Figure 2:**
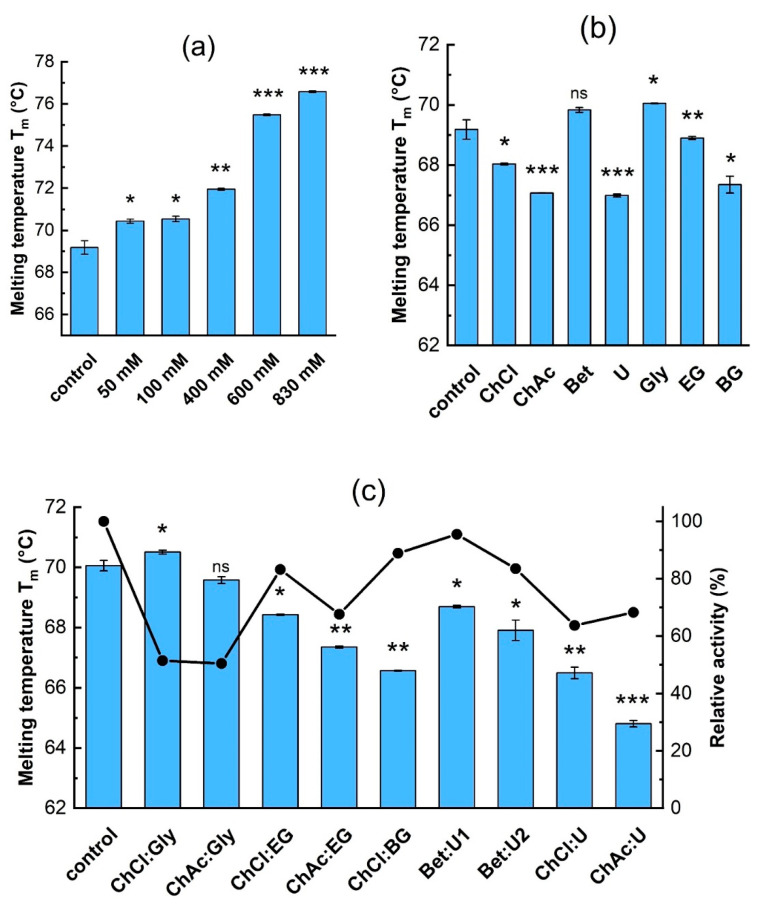
The effect of (**a**) lactose, and (**b**) individual DES components (concentrations are given in Table 2) on β-galactosidase thermal stability. (**c**) The effect of DES at 10% (*v*/*v*) on thermal stability and relative activity (that is, activity relative to the control) of the enzyme: ■—melting temperature, ●—relative activity. The control sample is the enzyme in 0.1 M acetate buffer, pH 5.5. Data are presented as mean ± SD of three independent experiments. The significance differences in T_m_ in comparison to the control sample given by the *t*-test are marked by asterisks with * *p* < 0.05, ** *p* < 0.01, *** *p* < 0.001, and ns means not significant.

Figure 2 shows the effect of individual components on the stability (represented by the melting temperature) and activity of β-galactosidase. Figures S1–S4 in the Supplementary Materials show the nanoDSF temperature ramps. First, the effect of reaction substrates on enzyme stability was studied. Increasing the lactose concentration enhanced the enzyme’s thermal stability by nearly 8 °C, from 69.2 °C (control sample in buffer) to 76.5 °C in the presence of 0.83 M lactose (Figure 2a). The onset temperature shifted accordingly, increasing from ≈59 °C to 66.8 °C (Table S1).

The stabilizing effect of lactose on β-galactosidase has also been observed by Illeová and Polakovič [41], who studied the thermal inactivation kinetics of the same enzyme in concentrated lactose solutions. They found that the thermal stability of the enzyme is about 5 °C higher when lactose is present. The high fluorescence of tyrosol interfered with the enzyme’s intrinsic fluorescence signal; consequently, only low concentrations of tyrosol (0.36–0.72 mM) could be used to determine its effect on β-galactosidase’s thermal stability. The results show no significant shift in T_m_ or T_onset_ (Table S1), indicating that such a low concentration of tyrosol has no effect on enzyme unfolding.

**Figure 3:**
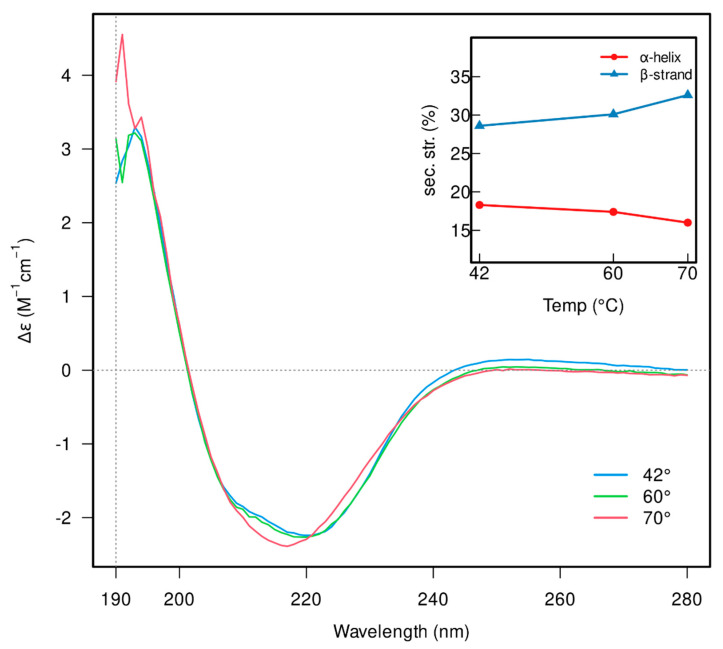
CD spectra of β-galactosidase recorded at 42 (blue), 60 (green) and 70 °C (red). The inset indicates the changes in the secondary structure elements’ content (α-helices and β-strands).

The folding and thermal stability of β-galactosidase was also verified using CD-spectroscopy (Figure 3). CD spectra measured at 42 °C, which corresponds to the temperature of the activity measurements, showed that the β-galactosidase contains ≈18% α-helices and ≈39% β-sheets. Increasing the temperature to 60 °C, T_onset_, did not significantly affect the α-helical content, though a slight increase in β-strand content was detected. Heating to 70 °C, T_m_, led to a shift in the spectra between 208 and 222 nm, suggesting partial denaturation of the helical structures, as well as a further increase in β-strand content (inset in Figure 3 and Table S2); however, full denaturation of β-galactosidase did not occur, which is in agreement with the results obtained by nanoDSF. Unfortunately, the effect of substrates and DESs on the enzyme structure at the same concentrations as the activity assays could not be measured by CD spectroscopy due to interferences with the protein signal.

Figure 2b shows the effect of individual DES components on the thermal stability and activity of β-galactosidase. Besides the values of T_m_ displayed in Figure 2b, nanoDSF temperature ramp measurements are shown in Figures S3 and S4 and the calculated values of T_m_ and T_onset_ are shown in Table S1. The DES components exhibited a different influence on the thermal stability of β-galactosidase. Among the hydrogen bond acceptors, betaine had no significant effect, while choline chloride and acetate decreased it by about 1.6 °C and 2.7 °C, respectively. Urea had the most pronounced destabilizing effect of all HBDs, causing a decrease in T_m_ by about 2.7 °C.

For the polyols glycerol, ethylene glycol, and butylene glycol, greater numbers of hydroxyl groups in a given molecule seemed to provide greater stabilization: in glycerol, the T_m_ increased by about 0.5 °C, while the diols either showed little effect (EG), or actually destabilized the molecule (the T_m_ decreased to 67.4 °C in BG; Figure 2b). Incubating the enzymes in solutions with the individual DES components for 24 h before measurement had no noticeable effect on the measured T_m_ (Figures S3 and S4); so, it appears that incubation time has no effect on the thermal stability of this enzyme.

Figure 2c shows the effect of adding complete DES mixtures at 10% (*v*/*v*) on the thermal stability of the enzyme. Comparing the thermal stability results of the individual DES components (Figure 2b) with those of the complete mixtures (Figure 2c) shows that there are both positive and negative synergistic effects on enzyme stability. The stabilizing effects of betaine, glycerol, and ethylene glycol outweighed the destabilizing influence of their respective counterparts within the corresponding DES. Moreover, betaine was able to effectively counteract the destabilizing effect of urea.

In the Bet:U1 solvent, where betaine was more prevalent, the enzyme’s T_m_ was correspondingly higher than in Bet:U2. A similar trend was observed in the DESs containing glycerol and ethylene glycol, which effectively mitigated the destabilizing effects of choline chloride and choline acetate. Notably, the presence of the ChCl:Gly combination led to an increase in T_m_ by approximately 1 °C. The destabilizing effects of choline chloride, choline acetate, urea, and butylene glycol persisted or were even amplified when combined in a DES. The combination of choline chloride and butylene glycol resulted in a T_m_ decrease to 66.6 °C, which is approximately 1.5 °C and 1 °C lower than in the presence of choline chloride and butylene glycol individually. A similar effect was observed for the ChAc:U combination, where T_m_ dropped to 64.8 °C.

Figure 2c also shows the effect of DES addition on relative activity. It should be noted that enzyme activity was measured at a high lactose concentration and in the presence of tyrosol. The results show that the enzyme’s activity exhibits the same trend as its stability upon the addition of urea-based DESs. Decreases in activity were observed when polyol-based DESs were used, which could arise from inhibition or inactivation of the enzyme. In particular, the glycerol-based DESs ChCl:Gly and ChAc:Gly significantly reduced enzymatic activity, although they had the strongest stabilizing effect.

The relationship between enzyme activity and structure is not straightforward. While structural changes due to the presence of DES may lead to lower stability, we hypothesize that they do not substantially affect the active site, with the exception of polyols. Polyols are galactosyl acceptors and could bind to the active site, thereby inhibiting the enzyme activity. Inhibition of β-galactosidase by glycerol and ethylene glycol was observed by Irazoqui et al. [20], who studied the transglycosylation activity of β-galactosidase from *A. oryzae*. In contrast, the activity of *Saccharomyces cerevisae* β-fructofuranosidase was highest in DESs containing EG as the HBD [42], although when DES concentration exceeded 40% (*v*/*v*), a complete halt in transfructosylation of tyrosol was observed. Unlike our findings, Hoppe et al. [35] reported increased activity of *Kluyveromyces lactis* β-galactosidase with ChAc-based DESs, showing 270% activity with 5% ChAc:Gly. In contrast, ChCl:Gly reduced activity to ~55%. These differences may stem from their use of a different enzyme or the *o*NPG substrate.

Wu et al. [43] observed effects similar to ours when examining ChCl- and ChAc-based DESs on horseradish peroxidase. Additionally, they found that DESs influenced the enzyme’s secondary structure; the enzyme possessed greater α-helix content and lower β-sheet and random coil contents in the presence of ChCl-based DESs compared to ChAc-based ones. These findings highlight the complex and variable impact of DESs on enzymes, which depends on factors like enzyme source, buffer, pH, substrate, and acceptor specificity, underscoring the need for a case-by-case analysis.

### 3.3. Effect of DESs on Enzyme’s Specificity Towards Galactosyl Acceptors

**Figure 4:**
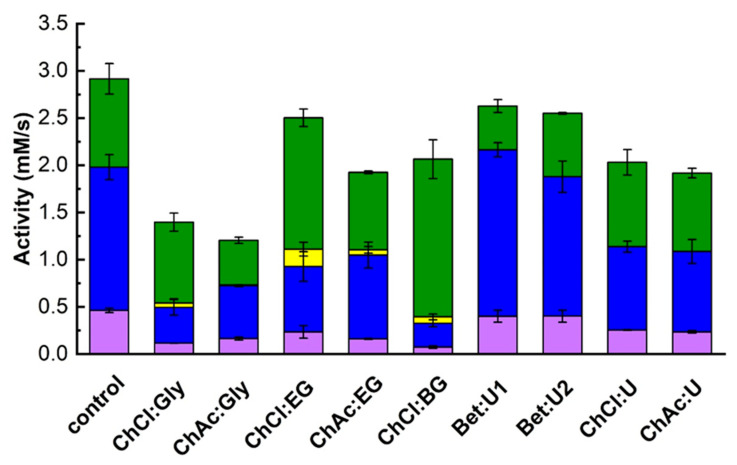
The effect of DES on the transglycosylation and hydrolytic activities (Equations (17)–(19)). Reaction conditions: 830 mM lactose, 72.4 mM tyrosol, 10% (*v*/*v*) DES, 50 µL of 1 g/L enzyme stock solution, pH 5.5, 42 °C; DES free system taken as control. Color codes: ■—tyrosol, ■—lactose, and ■—polyols transglycosylation activities, ■—hydrolytic activity.

The effect of DES addition on the specificity of β-galactosidase toward galactosyl acceptors was examined by measuring transglycosylation and hydrolytic activities. As shown in Figure 4, the presence of DESs influenced both activity types. To evaluate the statistical significance compared to control samples, a *t*-test was performed for each condition, and the *p*-values are presented in Table S3. Transglycosylation activities ($a_{GOS}$ and $a_{TG}$) decreased in nearly all cases except when Bet:U1 or Bet:U2 were used, indicating that betaine-based DESs helped retain activity. In contrast, DESs containing polyols, particularly those based on ChCl, exerted a significantly negative impact on transglycosylation. Meanwhile, hydrolytic activity was greatly enhanced by ChCl:EG and ChCl:BG. Additionally, small amounts of polyol galactosides (GlyGal, EGGal, BGGal) formed in these conditions, suggesting that DESs also affect acceptor utilization.

Relative activities (Table 3), calculated from the data in Figure 4, were used to analyze changes in enzymes specificity. All polyol-based DESs increased the enzyme’s affinity for water as a galactosyl acceptor ($a_{hydro}$), as evidenced by increased galactose release. Simultaneously, except for ChCl:Gly, these DESs reduced the enzyme’s preference for both tyrosol and lactose, leading to lower relative transglycosylation activity.

A comparison between DESs based on ChCl and those based on ChAc further highlighted the influenced of the HBA component. ChCl:polyol DESs caused a marked drop in transglycosylation activity while increasing hydrolytic activity, in contrast to ChAc:polyol DESs, which preserved a more balanced activity profile. On the other hand, betaine-based DESs (Bet:U1 and Bet:U2) retained enzyme specificity toward tyrosol, slightly increased lactose transglycosylation, and reduced hydrolytic activity. This suggests that betaine may stabilize the enzyme conformation conducive to transglycosylation, making these DESs promising for improved transglycosylation yields.

Our observations align with a growing body of literature emphasizing the variable and sometimes contrasting effects of DESs on glycoside hydrolase activity. For example, Weiz et al. [44] found that polyol-based DESs such as ChCl:Gly and ChCl:EG enhanced deglycosylation by 6-O-α-rhamnosyl-β-glucosidase, while urea-based DESs reduced activity. This parallels our finding that ChCl:EG and ChCl:BG improved hydrolytic activity, but urea-based DESs did not offer similar benefits. Furthermore, the authors observed improved activity when glycerol or ethylene glycol were used as co-solvents, supporting our hypothesis that polyol components can enhance hydrolytic efficiency.

Similar results were reported by Fotiadou et al. [31], who demonstrated that glycols enhanced pNPG hydrolysis catalyzed by immobilized β-glucosidase. Hoppe et al. [35,45] also observed a 2- to 3-fold increase in β-galactosidase activity with the addition of glycerol or ethylene glycol, although they noted a decline in activity at higher concentrations (>20% *v*/*v*). While we did not investigate varying DES concentrations, our results similarly show that polyol-based DESs such as ChCl:EG and ChCl:BG significantly enhanced hydrolytic activity, suggesting that the presence of glycols—regardless of concentration—can positively influence enzyme function. Xu et al. [28] reported comparable findings, with improved hydrolysis of p-nitrophenyl-β-D-glucopyranoside by β-glucosidase in the presence of ChCl:EG and ChCl:PG, further supporting the activating role of polyol-based DESs in hydrolytic reactions.

While hydrolytic activity in DESs has been more extensively studied, the impact on transglycosylation remains underexplored. Sandoval et al. [46,47] investigated β-galactosidase from *Thermus thermophilus* and found that both ionic liquids and bio-solvents influenced regioselectivity and overall activity through non-specific interactions. These solvents induced conformational changes in the enzyme’s secondary and tertiary structure, leading to altered catalytic behavior. Notably, solvents derived from dimethylamide and glycerol shifted Biolacta β-galactosidase regioselectivity from β-(1→4) to β-(1→6) linkages [48,49]. These findings highlight the critical influence of solvent–protein interactions on enzymatic outcomes, reinforcing the idea that DES-induced structural changes may underlie the reduced transglycosylation we observed with polyol-based DESs.

Further evidence of the diverse impact of green solvents on transglycosylation comes from a study on transfructosylation by *S. occidentalis* β-fructofuranosidase, where most solvents negatively affected trisaccharide synthesis, with few exceptions. In another example, methanolysis of starch catalyzed by α-amylase from *T. maritima* in ChCl:urea DESs showed increased alcoholysis/hydrolysis ratios and a ~27% higher methyl-glycoside yield when methanol was added at 10% *v*/*v* to a 60% DES solution [50]. These studies suggest that modifying solvent composition can influence not only enzyme activity but also product distribution and selectivity.

Together, our results and the literature illustrate that the impact of DESs on enzyme activity, specificity, and regioselectivity is multifaceted and strongly influenced by the DES components and the enzyme’s structural response. For β-galactosidase, polyol-based DESs promote hydrolysis but hinder transglycosylation, while betaine-based DESs appear more suitable for selective transglycosylation reactions. These effects are likely mediated through alterations in enzyme conformation and hydration, underscoring the need for mechanistic studies that link DES structure to enzyme function. As such, DESs offer untapped potential for fine-tuning biocatalytic reactions, particularly transglycosylation, and warrant further investigation to harness their full capabilities in green chemistry and synthetic biology.

### 3.4. Effect of DESs on the Transglycosylation of Tyrosol

**Figure 5:**
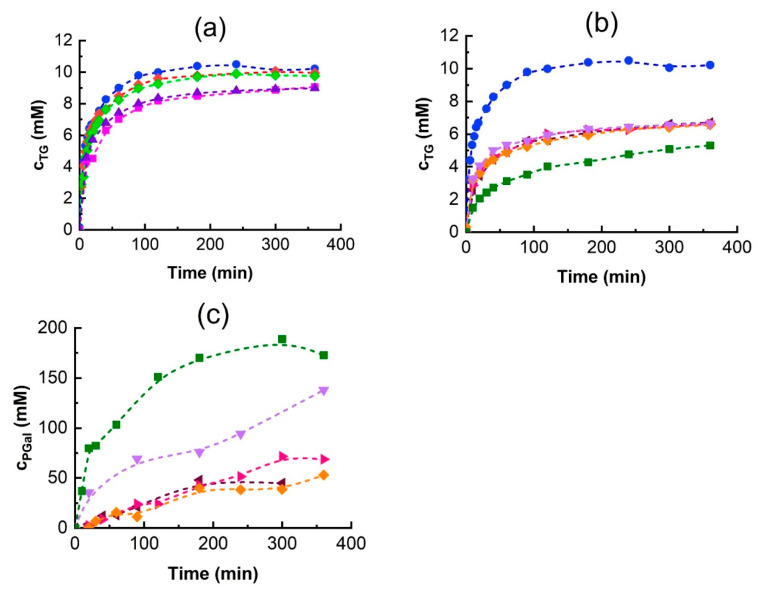

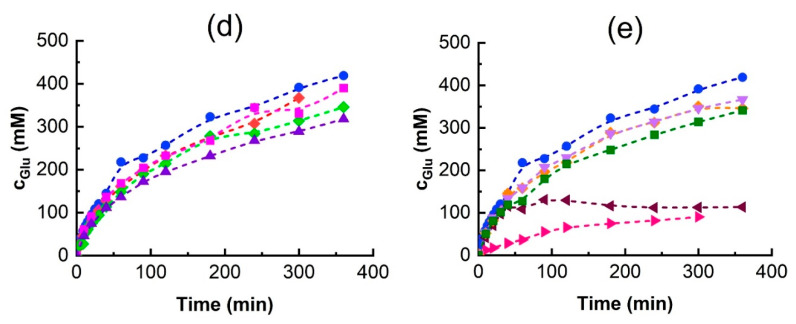
The effect of DES on the time course of the transglycosylation reaction over 6 h. TG formation upon addition of (**a**) urea and (**b**) polyol-based DESs, (**c**) PGal formation, and (**d**,**e**) glucose release. Reaction conditions are the same as in Figure 4 except that the 5 g/L enzyme stock solution was used. DES symbols: ●—control (without DES), ◆—Bet:U1, ◆—Bet:U2, ■—ChCl:U, ▲—ChAc:U, ◄—ChCl:Gly, ►—ChAc:Gly, ◆—ChCl:EG, ▼—ChAc:EG, ■—ChCl:BG. Panels (**a**,**d**) show the effects of ureas-based DESs while panels (**b**,**c**,**e**) show the effects of polyol-based ones.

To evaluate the effect of DES addition on TG production, reactions were conducted for 6 h in different DESs. The progress curves of TG, PGal and glucose are shown in Figure 5. The results of TG production in different DESs are in agreement with observations regarding the transglycosylation activity in Table 3. The addition of betaine-based DESs resulted in TG production comparable to that of the control. Similarly, ChCl:U and ChAc:U led to slightly lower TG yields than the control but remained comparable despite lowering the overall β-galactosidase activity by about 20%.

Polyol-based DESs significantly reduced TG production (Figure 5b): the addition of glycerol and ethylene glycol-based DESs decreased the final TG concentration to approximately 60% of the control, while the addition of ChCl:BG reduced it to 40%. The reduction in TG synthesis is due to the high formation of polyol galactosides (PGal), as shown in Figure 5c. Although $a_{PGal}$ was low, the extended reaction times led to PGal becoming the dominant product.

Among the tested DESs, ChCl:EG addition gave the highest $a_{PGal}$ activity (Figure 4), whereas the ChCl:BG mixture synthesized the most PGal over 6 h (Figure 5c). This explains the observed decrease in TG production. The finding that glycerol and ethylene glycol are good acceptors of the galactosyl moiety in the *A. oryzae* β-galactosidase transglycosylation reaction is corroborated by the results published by Irazoqui et al. [51], who reported high yields of the corresponding galactosides. The glycerol transglycosylation by *Kluyveromyces lactis* β-galactosidase was also reported; however, only low yields of the glycerol galactoside product were observed [52].

Karkeszová et al. [42] investigated the effect of DES addition on the synthesis of tyrosol β-D-fructoside by Saccharomyces cerevisiae β-fructofuranosidase. In their work, the highest product yield was achieved with the addition of ChAc:U, despite the fact that this DES had the worst impact on enzymatic activity. On the contrary, they observed that DESs composed from betaine and polyols enhanced the enzymatic activity the most, but the yield of tyrosol β-D-fructoside was the lowest. In that case, the probable cause was the competition of polyols with tyrosol for the fructosyl residue. In contrast to our results, the addition of 10% (*v*/*v*) of ChCl:BG enhanced the turn-over number of salidroside production by β-glucosidase by 55%, while the same DES had a negative effect on the hydrolysis of salicyl alcohol β-D-glucoside [31]. 

In transglycosylation reactions, product accumulation typically declines after reaching a maximum due to the increasing rate of hydrolysis surpassing the formation rate. Interestingly, in TG synthesis using β-galactosidase from *A. oryzae*, the TG concentration plateaued and remained unchanged over the experimental time (Figure 5a,b). One possible explanation is enzyme inhibition or inactivation. To determine whether the enzyme remained active, glucose release was monitored (Figure 5d,e). Generally, the glucose concentration increased over time, with its release rate being highest at the beginning of the reaction and then gradually decreasing, indicating that the enzyme was still active.

However, glycerol-based DESs had a different behavior. In ChAC:Gly, the rate of glucose release was very low from the beginning of the reaction, and in ChCh:Gly, after a rapid onset, the reaction completely stopped after approximately 2 h. These results are in excellent agreement with the hydrolytic activity measurements in Table 3. Interestingly, while the effect of Gly and EG-based DESs on TG synthesis was the same, their effect on glucose release was diametrically different (Figure 5b,e). Although Gly was shown to stabilize the enzyme, its negative effect on enzyme activity is likely due to inhibition.

### 3.5. Effect of Initial Tyrosol Concentration on Transglycosylation

**Figure 6:**
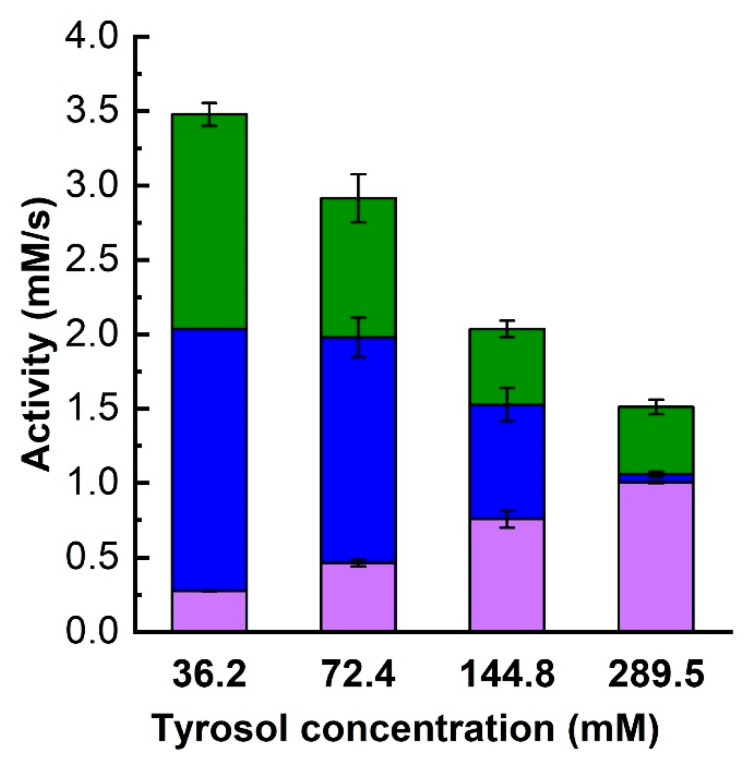
The effect of tyrosol concentration on the transglycosylation and hydrolysis activities of β-galactosidase. Reaction conditions are as in Figure 4. Color codes: ■—tyrosol, and ■—lactose transglycosylation activities, ■—hydrolytic activity.

In tyrosol β-D-galactoside synthesis, the desired galactoside acceptor is tyrosol, which can, itself, affect enzyme stability and activity. Since the effect of tyrosol on the enzyme’s thermal stability could not be assessed, its impact on the enzyme was evaluated by measuring changes to the enzyme’s activity. As an acceptor substrate, an increasing tyrosol concentration is expected to enhance the relative proportion of TG synthesis when substrate competition occurs between lactose, tyrosol, water, and other potential acceptors. To investigate this effect, initial reaction rate experiments were conducted without DESs but with varying tyrosol concentrations ranging from 36.2 to 289.5 mM. The results are shown in Figure 6.

It was observed that a higher initial tyrosol concentration enhanced the initial tyrosol transgycosylation activity, $a_{TG}$. At the same time, the overall enzyme activity decreased, indicating enzyme inhibition or inactivation. This activity decrease was accompanied by a significant decline in lactose hydrolysis and, especially, lactose transglycosylation. While the relative hydrolytic activity decreased only slightly, lactose transglycosylation was almost completely suppressed at a tyrosol concentration of 289.5 mM. This change in the ratio of tyrosol to lactose transglycosylation activities is summarized in Table 4. Since lactose is in molar excess relative to tyrosol in all cases, we hypothesize that such a significant suppression of GOS formation and therefore a change in enzyme specificity towards the acceptor might be due to a change in the secondary structure of β-galactosidase caused by tyrosol.

**Figure 7:**
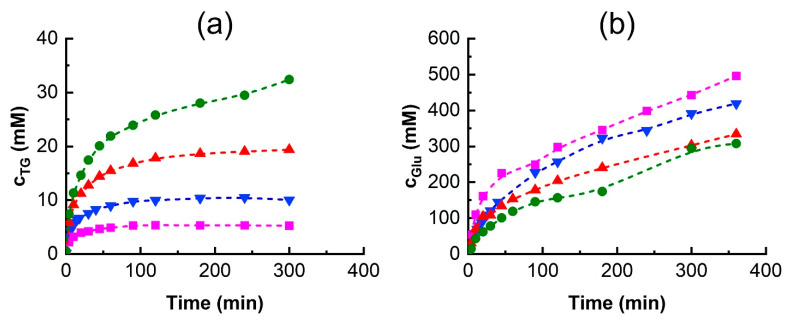
The effect of tyrosol concentration on the time course of the reaction over 6 h. (**a**) TG production and (**b**) glucose release with different initial tyrosol concentrations. Reaction conditions are as in Figure 4 except that the tyrosol concentration was ■—36.2 mM, ▼—72.4 mM, ▲—144.8 mM, and ●—289.5 mM.

Higher TG synthesis and lower overall activity at higher tyrosol concentrations were also seen in 6 h reactions, as shown in Figure 7. While increasing the tyrosol concentration resulted in higher TG amounts (Figure 7a), the decrease in overall activity was reflected in a decrease in the amount of glucose released and thus in a decrease in lactose conversion (Figure 7b). Figure 7b also shows that the reactions were still proceeding after 6 h of reaction time, suggesting that the effect of tyrosol may be inhibitory rather than inactivating. The final TG concentration at an initial tyrosol concentration of 289.5 mM reached nearly 33 mM (Figure 7a), representing an 11% molar yield. This yield is slightly lower than the 14% yield achieved with 36.2 mM tyrosol. However, despite the somewhat lower product yield, a higher TG concentration is advisable from a process perspective as it is advantageous for downstream processing.

In our previous results with immobilized *Aspergillus oryzae* β-galactosidase, Hollá et al. [22] observed that a higher initial tyrosol concentration resulted in lower lactose conversion pointing to possible substrate inhibition, and we also observed a similar tyrosol effect when studying tyrosol transfructosylation by *Saccharomyces cerevisiae* β-fructofuranosidase [42]. Likewise, Qi et al. [53] reported a decrease in yield once the initial tyrosol concentration surpassed 250 mM. In their research, they obtained a 50% yield of total tyrosol glycosides from *Enterobacter cloacae* B5 β-galactosidase when the initial concentrations of lactose and tyrosol were 1000 mM and 250 mM, respectively.

## 4. Conclusions

This study demonstrates that both substrate composition and deep eutectic solvents (DESs) significantly affect the activity, stability, and specificity of β-galactosidase from *Aspergillus oryzae* in tyrosol transglycosylation. Lactose stabilized the enzyme by increasing its melting temperature, while high tyrosol concentrations caused activity loss, likely due to substrate inhibition. Individual DES components like betaine and glycerol enhanced stability, but their combinations did not consistently improve enzymatic performance. DESs also altered enzyme specificity and, in some cases, increased hydrolytic activity.

Structural analyses via CD spectroscopy and nanoDSF confirmed that the enzyme retained its fold in the presence of DESs and substrates, with partial unfolding observed upon heating. Due to overlapping hydrolytic and transglycosylation activity, product monitoring was identified as a more reliable indicator of performance than total activity. These findings emphasize the importance of empirical optimization and methodological care in enzyme applications involving DESs.

## Figures and Tables

**Table 1:** List of DESs used in this study, their abbreviations, densities and viscosities at 25 °C.

HBA	HBD		Molar Ratio HBA:HBD	Abbreviation	Density (g/cm^3^)	Viscosity (mPas)	Reference
Betaine	Urea	Water	1.44:1:2.76	Bet:U1	1.17	NA	-
	Urea	Water	1:2:1.33	Bet:U2	1.19	NA	-
Choline chloride	Glycerol		1:2	ChCl:Gly	1.19	376.0	[34,35]
	Ethylene glycol		1:2	ChCl:EG	1.12	52.1	[34,35]
	Butylene glycol		1:4	ChCl:BG	1.04	88	[36]
	Urea		1:2	ChCl:U	1.18	750	[35,37]
Choline acetate	Glycerol		1:2	ChAc:Gly	1.17	284.6 *	[35]
	Ethylene glycol		1:2	ChAc:EG	1.10	37.6 *	[35]
	Urea		1:2	ChAc:U	1.18	179.4 *	[35]

NA—data not available, *—viscosity at 26 °C.

**Table 2:** Concentrations of substrates and individual DES components in measurements of thermal denaturation of β-galactosidase.

Component	Concentration (mM)
Tyrosol	0.36 or 0.72
Lactose	50, 100, 400, 600, 830
Choline chloride	469.9
Choline acetate	415.4
Betaine	602.7
Urea	909.1
Glycerol	735.1
Ethylene glycol	845.8
Butylene glycol	652.5

**Table 3:** The effect of DES on the relative activities of transglycosylation of tyrosol and lactose, and lactose hydrolysis. Relative activity is expressed as the ratio of a given enzyme activity to the sum of all activities shown in Figure 4.

DES	Relative Activity		
	$a_{TG}$ (%)	$a_{GOS}$ (%)	$a_{hydro}$ (%)
Control	16	52	32
ChCl:Gly	8	27	61
ChAc:Gly	14	46	39
ChCl:EG	9	28	56
ChAc:EG	8	46	43
ChCl:BG	4	12	81
Bet:U1	15	67	18
Bet:U2	16	58	26
ChCl:U	12	43	44
ChAc:U	12	44	43

**Table 4:** The effect of tyrosol concentration on the relative activities of transglycosylation of tyrosol and lactose, and lactose hydrolysis. Relative activity is expressed as the ratio of a given enzyme activity to the sum of all activities shown in Figure 4.

Tyrosol (mM)	Relative Activity		
	$a_{TG}$ (%)	$a_{GOS}$ (%)	$a_{hydro}$ (%)
36.2	8	51	41
72.4	16	52	32
144.8	37	38	25
289.5	66	4	30

## Data Availability

Data will be made available on request from the corresponding author.

## Supplementary Materials

The following supporting information can be downloaded at: https://www.mdpi.com/article/10.3390/biom15060801/s1, Figure S1: Illustrative chromatograms of HPLC analyses of substrates and products of transglycosylation reactions; Figure S2 NanoDSF measurements of β -galactosidase unfolding in the presence of lactose and tyrosol; Figure S3: NanoDSF measurements of β-galactosidase unfolding in the presence of hydrogen bond acceptors; Figure S4: NanoDSF measurements of β-galactosidase unfolding in the presence of hydrogen bond donors; Figure S5: NanoDSF measurements of β-galactosidase unfolding in the presence of 10% (*v*/*v*) DESs. The control sample is the enzyme in 0.1 M acetate buffer, pH 5.5; Table S1: The results of nanoDSF measurements showing the effect of substrates, DES components and 10% (*v*/*v*) DES on the onset and melting temperatures of β-galactosidase; Table S2: Secondary structure composition of β-galactosidase at 42, 60 and 70°C as deconvoluted by CONTINLL. The CD signal was taken between 190–240 nm; Table S3: *p*-values expressing the statistical significance of the effect of 10% (*v*/*v*) DES on the activity of tyrosol transglycosylation, *a_TG_*, lactose transglycosylation, *a_GOS_*, and hydrolytic activity, *a_hydro_*, compared to the activities of the control sample.
