# Supplementary material for: β-Galactosidase-Catalyzed Transglycosylation of Tyrosol: Substrates and Deep Eutectic Solvents Affecting Activity and Stability
Source: Biomolecules. 2025 May 31;15(6):801. doi: 10.3390/biom15060801 (PMC12191228; doi:10.3390/biom15060801)
Supplement: Supplementary file 1 [file biomolecules-15-00801-s001.zip › biomolecules-3624824-supplementary.pdf]

β-Galactosidase-catalysed transglycosylation of tyrosol: substrates and deep eutectic solvents affecting activity and stability

Alžbeta Koššuthová[1], Monika Antošová[1], Vladena Bauerová-Hlinková[2], Jacob A. Bauer[2], Milan Polakovič[1*]

Supplementary material

A

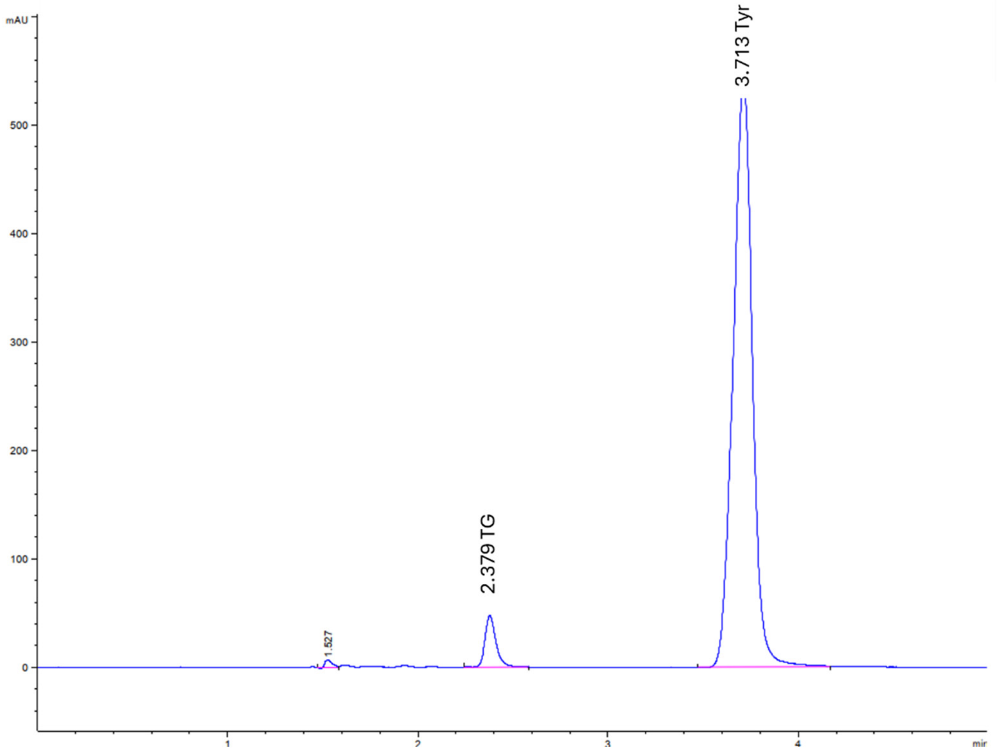

Figure S1 (A). Illustrative chromatograms of HPLC analyses of tyrosol and tyrosol β-D-galactoside on Zorbax Eclipse XDB C-18 column.

B

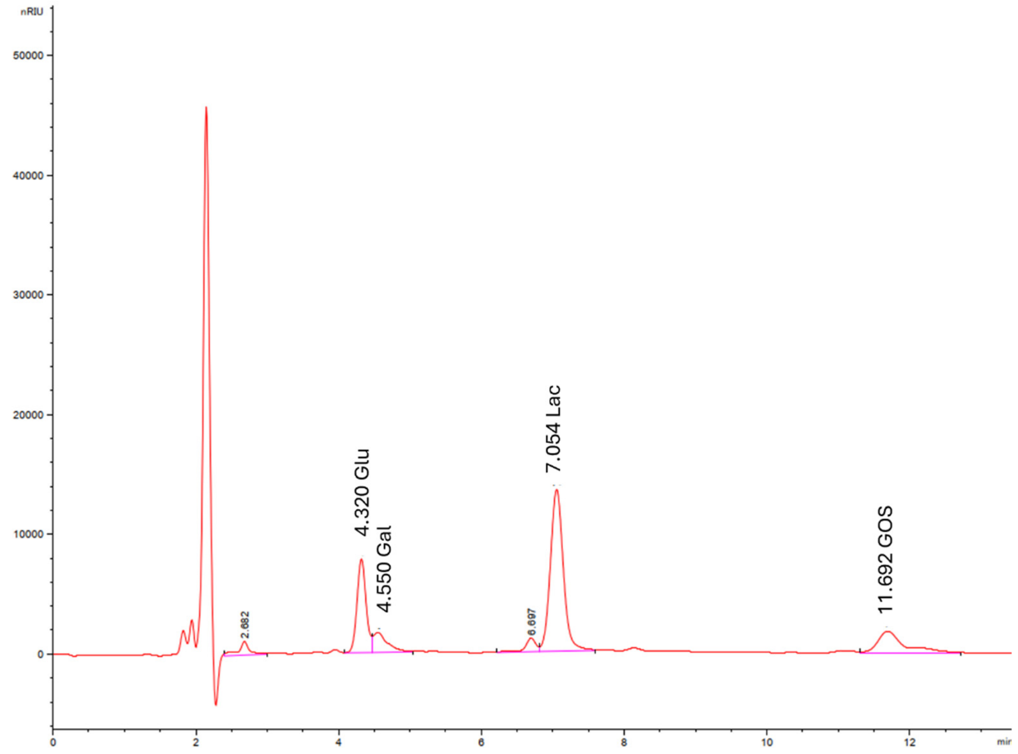

C

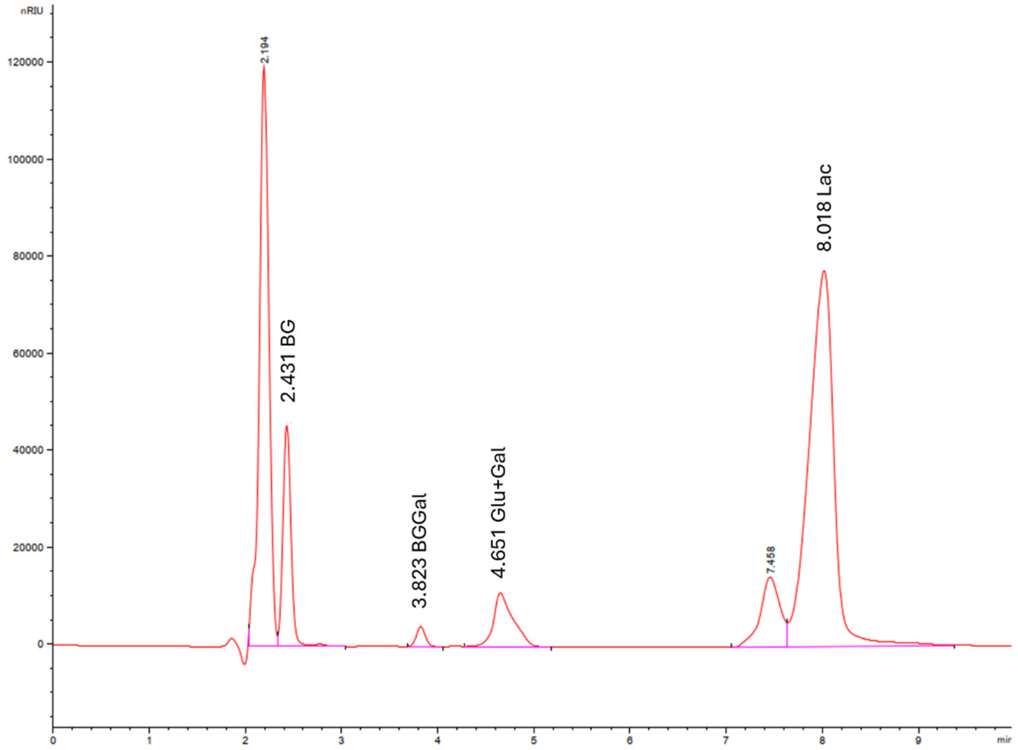

Figure S1 (B and C). Illustrative chromatograms of HPLC analyses of substrates and products of transglycosylation reactions on Luna Omega Sugar column. The samples are actual reaction mixtures without (B) or with the addition of DES ChCl:BG (C).

D

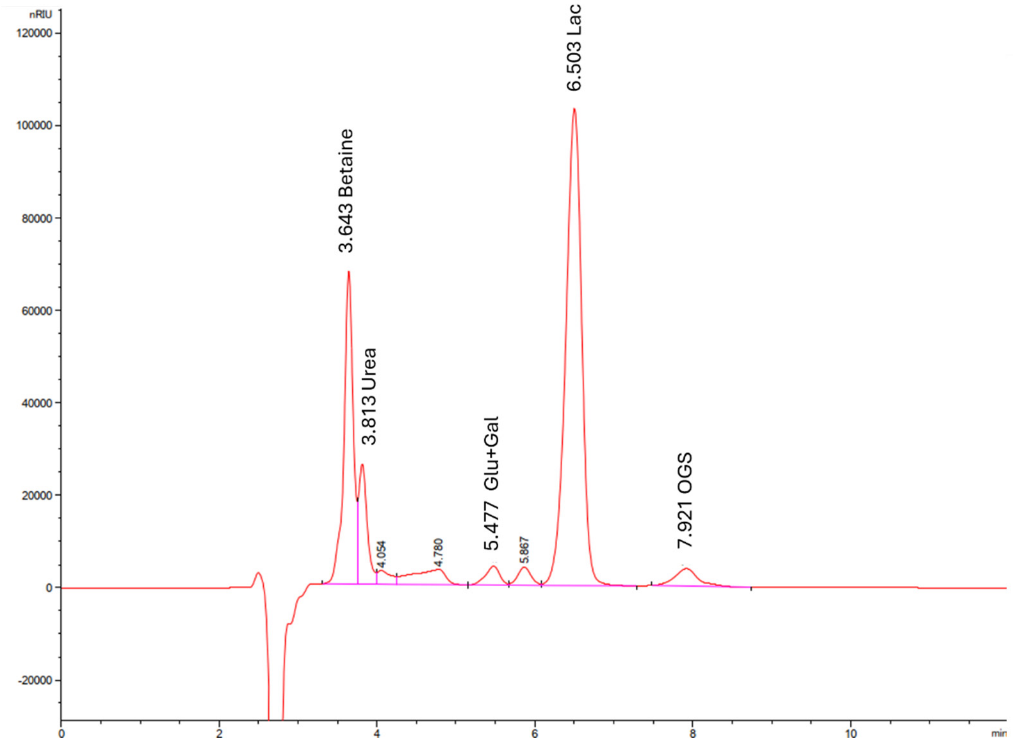
nRIU
120000
100000
80000
60000
40000
20000
0
-20000
0
2
4
6
8
10
min
3.643 Betaine
3.813 Urea
4.054
4.780
5.477 Glu+Gal
5.867
6.503 Lac
7.921 OGS

E

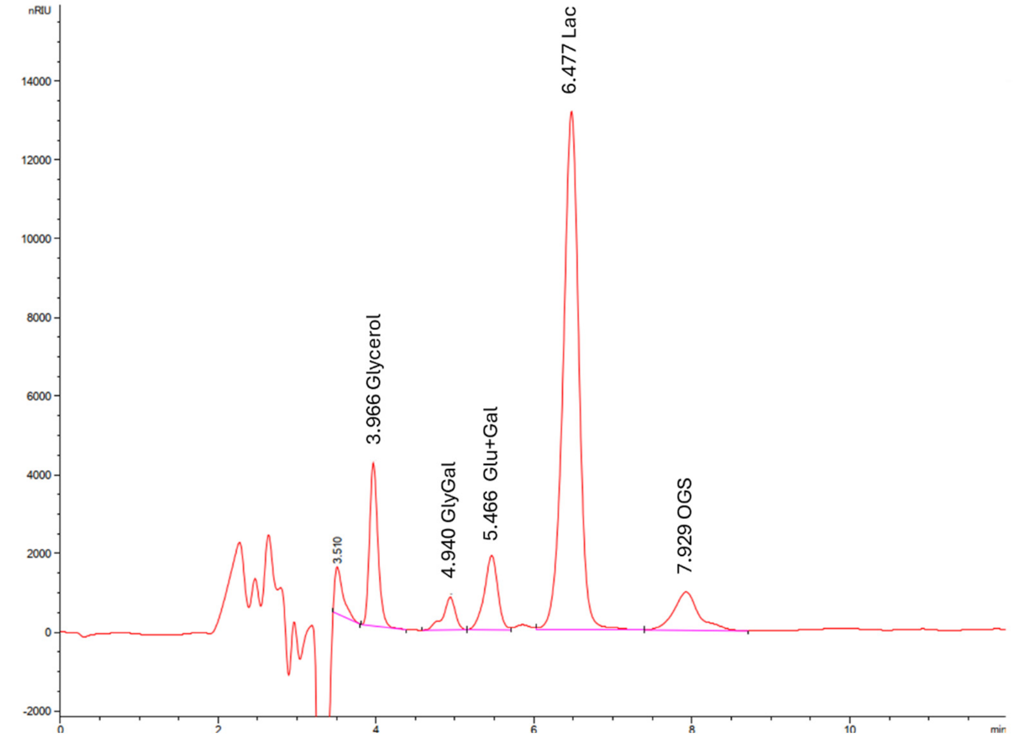
nRIU
14000
12000
10000
8000
6000
4000
2000
0
-2000
0
2
4
6
8
10
min
3.510
3.966 Glycerol
4.940 GlyGal
5.466 Glu+Gal
6.477 Lac
7.929 OGS

F

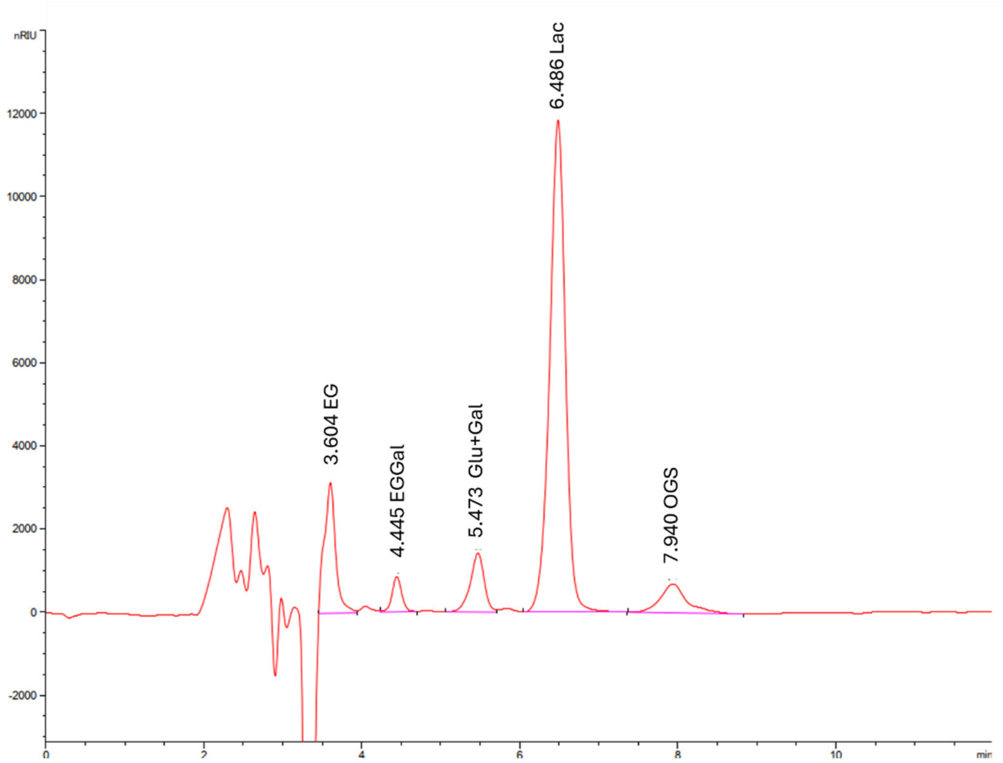

G

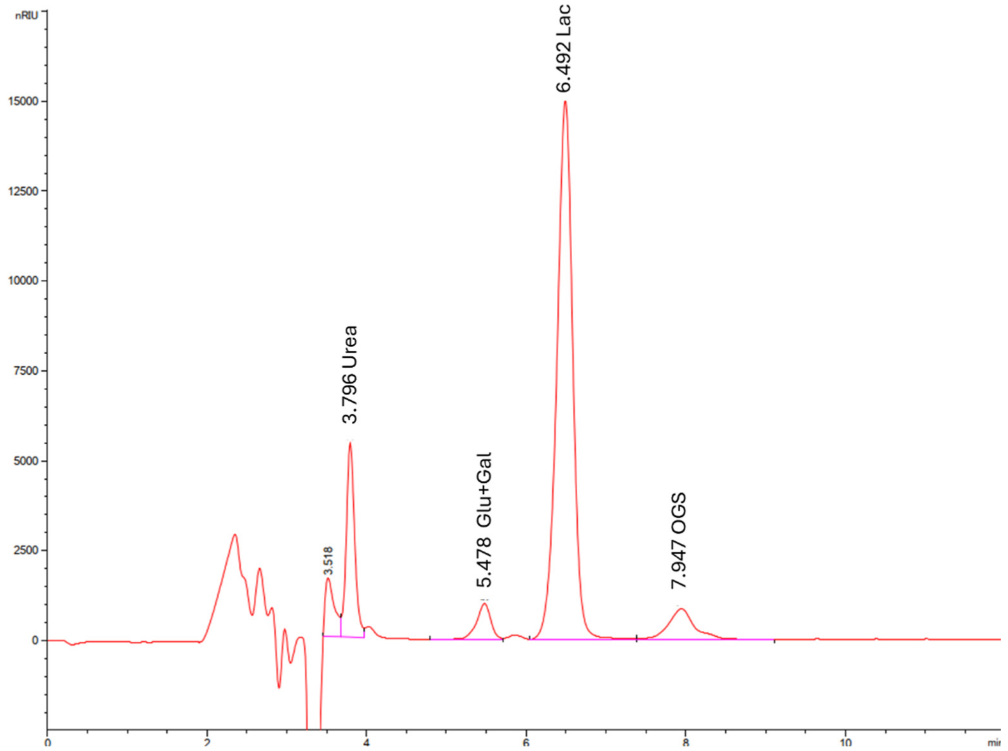

Figure S1 (D-G). Illustrative chromatograms of HPLC analyses of substrates and products of transglycosylation reactions on Asahipak NH2P-50 4E column. The samples are actual reaction mixtures with the addition of DESs Bet:U1 (D), ChCl:Gly (E), ChCl:EG (F) and ChCl:U (G).

A

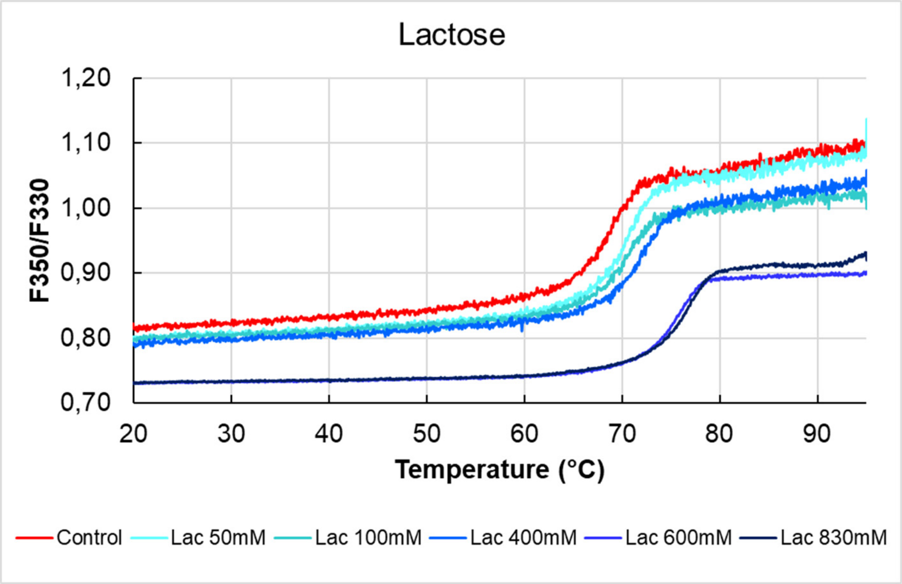

B

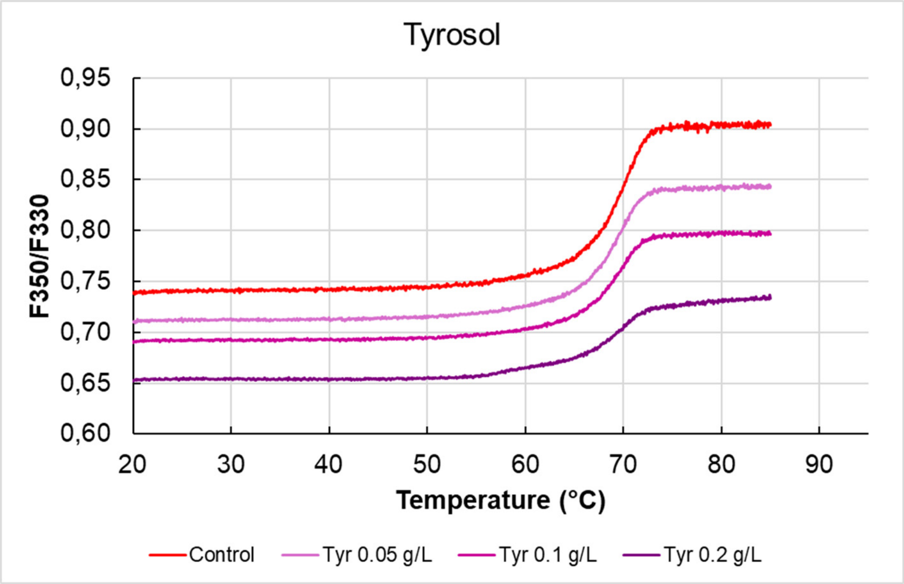

Figure S2. NanoDSF measurements of β-galactosidase unfolding in the presence of (A) lactose and (B) tyrosol, The control sample is the enzyme in 0.1 M acetate buffer, pH 5.5.

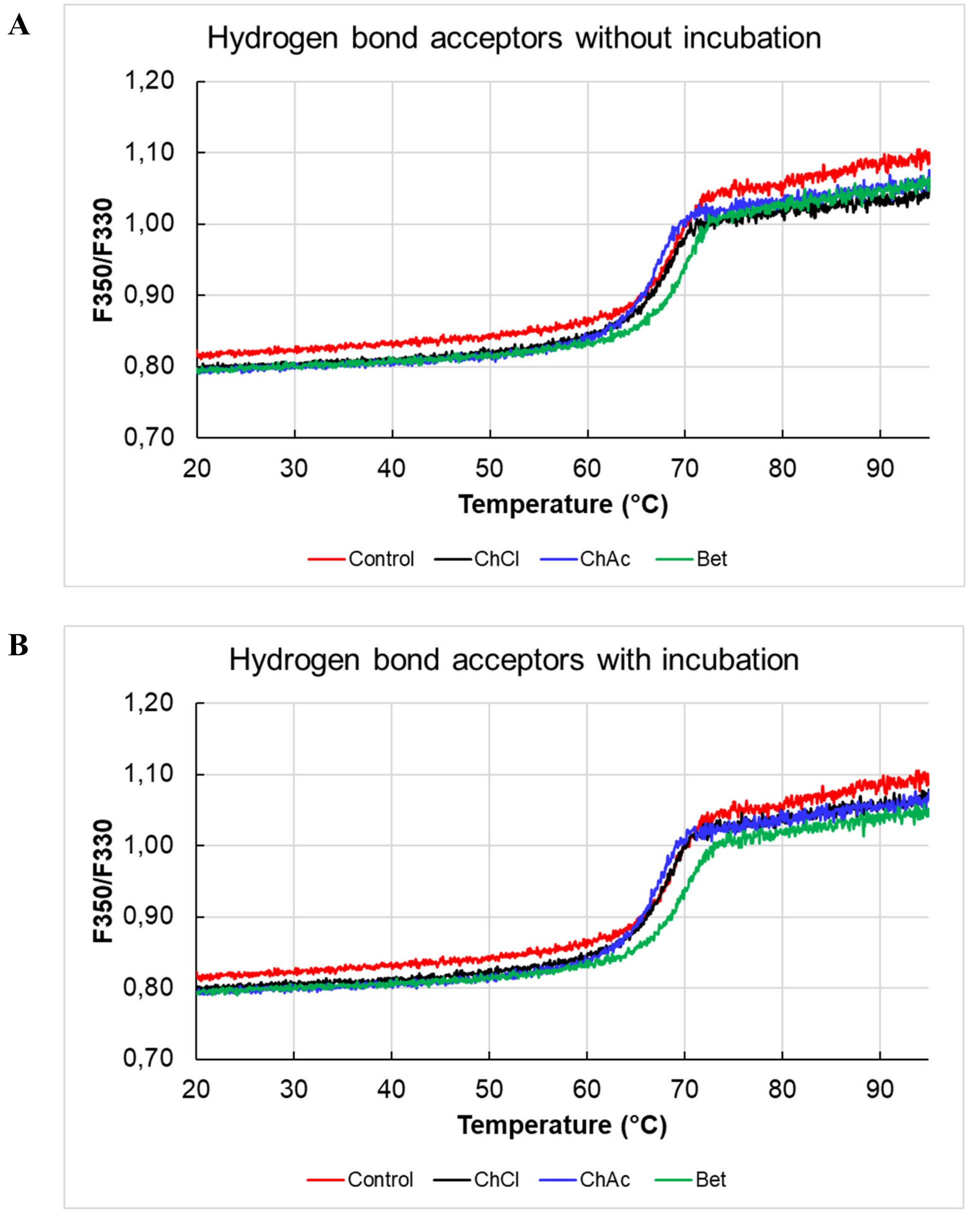

Figure S3. NanoDSF measurements of β-galactosidase unfolding in the presence of hydrogen bond acceptors (A) without pre-incubation, and (B) with 24 h pre-incubation. The control sample is the enzyme in 0.1 M acetate buffer, pH 5.5.

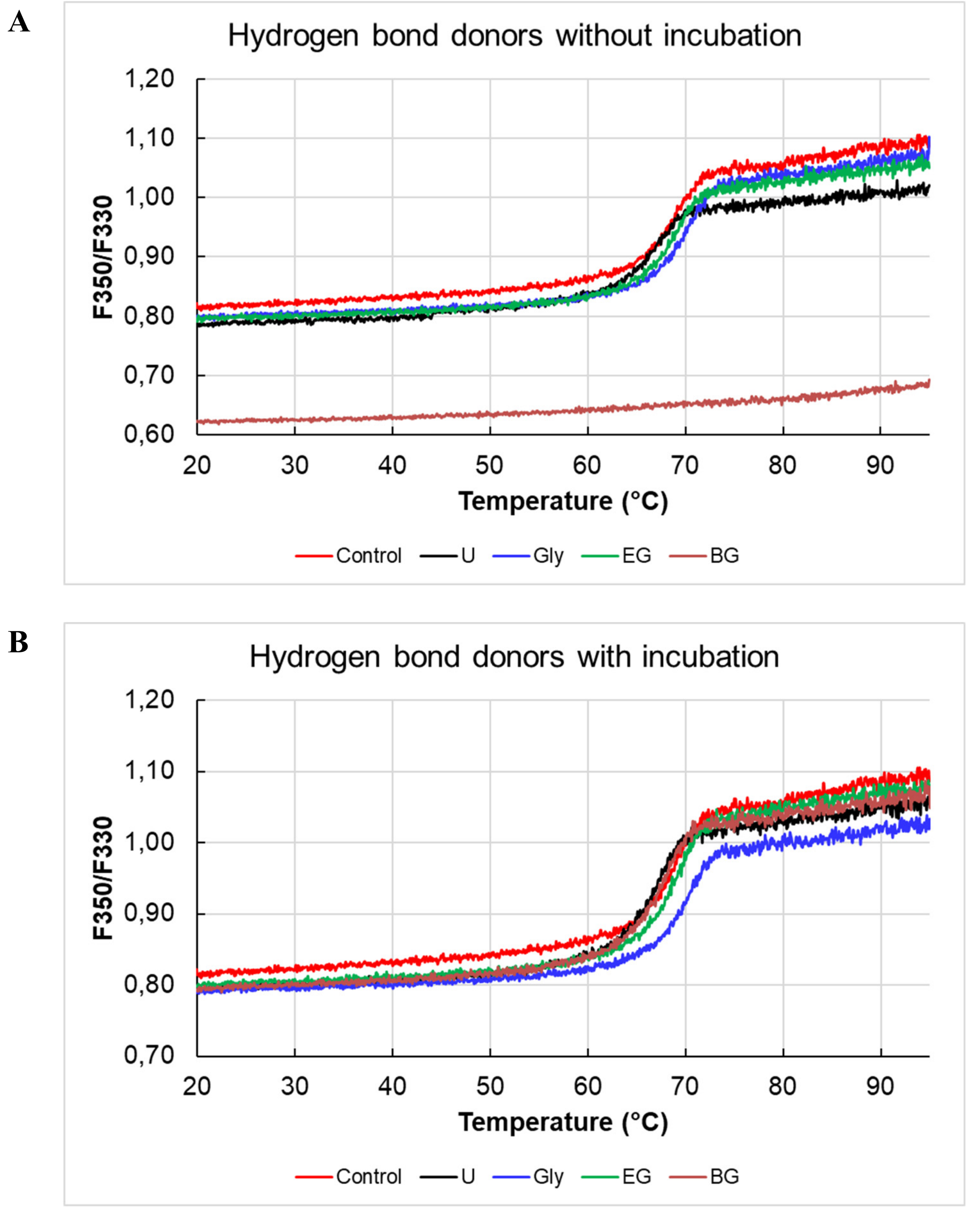

Figure S4. NanoDSF measurements of β-galactosidase unfolding in the presence of hydrogen bond donors (A) without pre-incubation, and (B) with 24 h pre-incubation. The control sample is the enzyme in 0.1 M acetate buffer, pH 5.5.

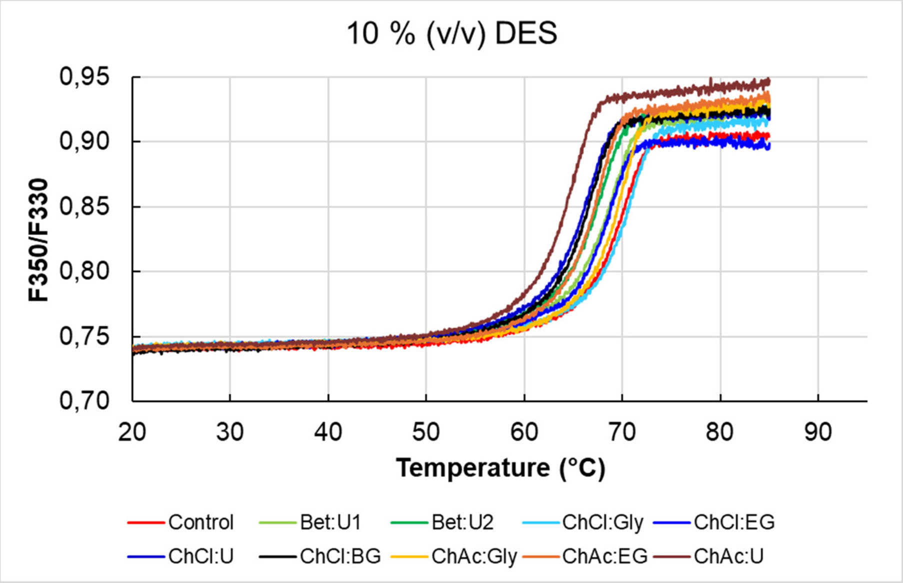

Figure S5. NanoDSF measurements of β-galactosidase unfolding in the presence of 10% (v/v) DESs. The control sample is the enzyme in 0.1 M acetate buffer, pH 5.5.

Table S1 The results of nanoDSF measurements showing the effect of substrates, DES components and 10% (v/v) DES on the onset and melting temperatures of β-galactosidase.

	T_{onset} (°C)	T_m (°C)
**Control**	61 ± 1	69.7 ± 0.2
**Concentration (mM)**	**Lactose**	
50	60.2 ± 0.3	70.4 ± 0.1
100	60.7 ± 0.5	70.5 ± 0.1
400	63.0 ± 0.3	71.95 ± 0.05
600	66.0 ± 0.1	75.48 ± 0.04
830	66.8 ± 0.1	76.58 ± 0.04
**Concentration (mM)**	**Tyrosol**	
0.36	61.5 ± 0.4	69.4 ± 0.2
0.72	61.1 ± 0.5	69.48 ± 0.05
**Deep eutectic solvent components**		
Choline chloride	56.8 ± 0.1	68.03 ± 0.03
Choline acetate	56.5 ± 0.3	67.07 ± 0.01
Betaine	60.7 ± 0.2	69.83 ± 0.08
Urea	56.5 ± 0.5	66.99 ± 0.05
Glycerol	60.9 ± 0.9	70.05 ± 0.01
Ethylene glycol	59.4 ± 0.1	68.90 ± 0.05
Butylene glycol	58 ± 1	67.4 ± 0.3
**Deep eutectic solvents**		
ChCl:Gly	61.24 ± 0.06	70.50 ± 0.06
ChAc:Gly	59.8 ± 0.4	69.6 ± 0.1
ChCl:EG	59 ± 1	68.42 ± 0.02
ChAc:EG	57.0 ± 0.1	67.35 ± 0.04
ChCl:BG	56.2 ± 0.2	66.56 ± 0.02
Bet:U1	59.3 ± 0.1	68.69 ± 0.04
Bet:U2	57.9 ± 0.9	67.9 ± 0.3
ChCl:U	55.6 ± 0.3	66.5 ± 0.2
ChAc:U	53.8 ± 0.2	64.8 ± 0.1

Table S2. Secondary structure composition of β-galactosidase at 42, 60 and 70°C as deconvoluted by CONTINLL. The CD signal was taken between 190–240 nm

	**42°C**	**60°C**	**70°C**
**Helical structures**	18.3%	17.4%	16.0%
**β-sheets**	28.6%	30.1%	32.6%
**others**	53.1%	52.6%	51.4%

Table S2 *p*-values expressing the statistical significance of the effect of 10% (v/v) DES on the activity of tyrosol transglycosylation, a_{TG}, lactose transglycosylation, a_{GOS}, and hydrolytic activity, a_{hydro}, compared to the activities of the control sample.

**DES**	**a_{TG} (mM/s)**	**a_{GOS} (mM/s)**	**a_{hydro} (mM/s)**
ChCl:Gly	< 0.001	0.002	0.046
ChAc:Gly	0.001	0.002	0.002
ChCl:EG	0.012	0.008	0.030
ChAc:EG	< 0.001	0.014	0.014
ChCl:BG	< 0.001	0.001	0.022
Bet:U1	0.160	0.081	0.003
Bet:U2	0.161	0.415	0.004
ChCl:U	0.001	0.008	0.095
ChAc:U	0.001	0.011	0.021
